# Impact of digital-based online physical education on undergraduate students’ physical and psycho-emotional wellbeing

**DOI:** 10.3389/fpsyg.2026.1885457

**Published:** 2026-08-11

**Authors:** Anna Prikhodko, Inna Bodrenkova, Tetiana Moshenska, Ihor Prykhodko

**Affiliations:** 1 Department of Physical Education, Yaroslav Mudryi National Law University, Kharkiv, Ukraine; 2 Department of Dance, Sports, and Choreography, Kharkiv State Academy of Physical Culture, Kharkiv, Ukraine; 3 Research Laboratory of Psychological Support of Service and Combat Activities of the National Guard of Ukraine, Educational and Scientific Institute for Personnel Work, National Academy of the National Guard of Ukraine, Kharkiv, Ukraine; 4 Department of Practical Psychology and Innovative Health Technologies, Educational and Scientific Institute “Ukrainian Engineering and Pedagogical Academy”, V. N. Karazin Kharkiv National University, Kharkiv, Ukraine

**Keywords:** digital pedagogy, frontline zone, mental health, multimodal approach, online learning, physical education, undergraduate students

## Abstract

**Background:**

The ongoing conflict in Ukraine has forced higher education institutions in frontline regions to transition to long-term remote learning, significantly increasing the risk of sedentary behavior and psychological stress among undergraduate students. Consequently, traditional physical education programs face critical organizational and pedagogical challenges within this exclusively online format.

**Objectives:**

This study aimed to evaluate the effectiveness of a 15-week comprehensive online physical education course integrating digital platforms and interactive tools on the physical activity levels and psycho-emotional wellbeing of undergraduate students at a frontline university.

**Methods:**

A single-group pretest-posttest design was employed with 735 first-year volunteers (361 males, 374 females). The intervention utilized a multimodal approach via Zoom and Moodle, incorporating digital quizzes, social media challenges, and diverse practical modalities (e.g., Tabata, Pilates). Assessments included the International Physical Activity Questionnaire (IPAQ-SF), Body Mass Index (BMI), and the “Well-being, Activity, and Mood” (WAM) questionnaire.

**Results:**

After 15 weeks, students showed a statistically significant increase in physical activity: females by 20.15 MET-min/week (*p* = 0.030) and males by 18.15 MET-min/week (*p* = 0.050). BMI values remained stable for the group. The most pronounced improvements were observed in the psycho-emotional sphere; notably, female students’ mood scores increased significantly by 1.2 points (*p* = 0.001). Overall, the group demonstrated positive dynamics across all WAM scales: wellbeing (+0.92%), activity (+1.33%), and mood (+1.56%).

**Conclusion:**

The integration of synchronous and asynchronous digital tools into physical education is associated with positive trends in counteracting hypodynamia and supporting mental health during periods of social isolation and conflict. This adaptive pedagogical approach could be recommended for universities and secondary educational institutions operating in distance or blended formats under crisis conditions.

## 1 Introduction

The global shift to online learning, accelerated by the COVID-19 pandemic, has significantly altered university students’ lifestyles, leading to a marked decline in daily energy expenditure and increased sedentary behavior (Aubert et al., 2022; Li et al., 2023; Chu and Li, 2022; Ha et al., 2025; Radu et al., 2020; Zakomorna et al., 2024). Greater screen time and less physical travel to campus have made sedentary behavior the dominant pattern among students (López-Valenciano et al., 2021; Neville et al., 2022; Romero-Blanco et al., 2020).

For Ukrainian higher education, these challenges were exacerbated by 4 years of full-scale war (Griban et al., 2024). In frontline regions, the situation has reached a critical point: constant threats of airstrikes, shelling, and psychological pressure have become a daily reality (Ogorenko et al., 2023; Savelyuk, 2022). These factors have a cumulative destructive impact on the psychoemotional state and physical health of educational participants (Kashkalda et al., 2023; Muntian and Poproshaiev, 2024; Rak et al., 2024). Under these conditions, transitioning to online learning via videoconferencing platforms (e.g., Zoom) has become the only way to ensure security while maintaining educational continuity.

Current research shows that organized physical activity is an essential means of promoting health, with the physiological aspect (training the autonomic nervous system) inextricably linked to the psychological aspect (development of self-regulation and resilience; Biddle et al., 2019; Joseph, 2025; Kolomiitseva et al., 2020; Mahindru et al., 2023). The proven correlation between high-intensity exercise and psychological wellbeing confirms that sport is not just a hobby but a necessary tool for developing cognitive control and emotional stability, enabling one to maintain concentration during periods of heightened danger (Joseph, 2025). In the context of distance learning and chronic stress, regular exercise and interactive fitness formats are becoming a decisive factor in overcoming social isolation and building high levels of individual resilience (Bodrenkova et al., 2024; Rak et al., 2024).

Online physical education is a specific and controversial format for a subject traditionally centered on physical activity in gyms or outdoors (Muntian et al., 2023; Petrova and Latyshev, 2025; Prikhodko, 2024). Despite objective limitations in realizing the developmental potential of physical education, modern information and communication technologies offer new opportunities to optimize the didactic process (Da-Wei et al., 2018; Petrova and Latyshev, 2025; Shepelenko et al., 2023; Valencia Pilamunga et al., 2026; Yefremenko et al., 2025). Under martial law, there is an urgent academic need to develop and implement adaptive pedagogical strategies that support physical activity in confined spaces.

Recent studies show that digital platforms (Moodle, Google Classroom), videoconferencing tools (Zoom, Meet), social media and video tutorials (YouTube, Instagram, TikTok), multimedia content, and sports apps are increasingly effective at promoting physical activity, providing methodological support, and expanding teaching opportunities (Da-Wei et al., 2018; Valencia Pilamunga et al., 2026; Petrova and Latyshev, 2025; Shepelenko et al., 2023; Yefremenko et al., 2025). Today’s youth actively use video tutorials and fitness trackers for physical activity and self-regulation (Petrova and Latyshev, 2025). Researchers highlight the benefits of digital tools for sports training and for promoting physical activity across diverse population groups (Asaulyuk et al., 2024; Bodrenkova et al., 2024; Cabral et al., 2022; Da-Wei et al., 2018). Online fitness programs and interactive challenges help address social isolation and physical inactivity in the context of distance learning (Rak et al., 2024; WHO, 2025a). At the same time, research on the effectiveness of comprehensive digital initiatives integrated directly into the physical education curriculum at universities in war zones remains fragmented (Muntian et al., 2023; Shepelenko et al., 2023; Yefremenko et al., 2025).

This study *aims* to evaluate the effectiveness of a comprehensive online physical education course on the physical and psychological wellbeing of Ukrainian undergraduate students at a university in a frontline zone. We hypothesize that integrating digital platforms, interactive tools, and traditional physical exercise can not only increase physical activity but also create a positive educational environment that supports the psychological and morpho-functional wellbeing of Ukrainian undergraduate students during the ongoing war.

## 2 Materials and methods

### 2.1 Participants

The study participants were healthy first-year undergraduate student volunteers (*n* = 735; 361 males, 374 females; mean age 18.33 ± 1.37 years, range 18–21 years), who studied online at different faculties at Yaroslav Mudryi National Law University located in the near-frontline region during the war. During the experiment, all participants lived in different regions of Ukraine or abroad as displaced persons. Medical examination records provided upon university admission confirmed that all participants were free of medical contraindications to physical activity of varying intensities. A single-group pretest-posttest design was employed to assess the targeted physical and psychological changes over 15 weeks. Given the absence of a control group, the primary analytical focus was the within-group comparison of baseline (September) and endpoint (December) indicators. All participants were informed of the study procedures and objectives, and were advised of their right to withdraw consent at any time without penalty. The study protocols adhered to the ethical standards for human experimentation established by the Helsinki Declaration.

### 2.2 Intervention

#### 2.2.1 Context and study design

“Physical Education” is a mandatory component of the undergraduate curriculum in Ukraine. At Yaroslav Mudryi National Law University, this course is delivered to first-year students over two semesters, with one 80-min session per week for 15 weeks. Given the security imperative to ensure student safety at a frontline university during the ongoing war, we transitioned the course to a fully online format on Zoom. To account for internet and power disruptions caused by the conflict, the course was structured flexibly. While synchronous Zoom attendance averaged 79%, the remaining participants completed the curriculum asynchronously via Moodle and social media platforms, achieving a total course completion rate of 93%. The sessions were facilitated by a team of eight highly qualified instructors, each possessing over 20 years of pedagogical experience; six of them have a PhD degree. The intervention combined structural oversight with participant autonomy. Synchronous sessions required active camera participation where safety permitted, while asynchronous completion was verified through video uploads of specific exercise routines on the student’s personal account on the Moodle platform, to the instructor’s corporate email, dedicated social media groups on Viber, WhatsApp, and Telegram, and weekly digital logs.

The 15-week study used a single-group longitudinal design. The program had its own Educational and Informational Electronic Complex (EIEC), hosted on the Moodle platform, and was enhanced by integrating a multimodal approach that included the following components: instructional design and modalities; digital integration; assessment and engagement.

#### 2.2.2 Instructional design and modalities

To compensate for the limitations of remote learning, a multimodal approach was adopted, balancing theoretical knowledge with practical motor skills. The theoretical segment (20–25 min) focused on physical literacy, health benefits of activity, nutrition, and psychoregulation, etc. The practical segment (55–60 min) included diverse activities adaptable to online learning, such as gymnastics, aerobics, Pilates, stretching, CrossFit, and Tabata training.

#### 2.2.3 Digital integration

The program employed a wide array of synchronous and asynchronous tools, including Moodle, Google Forms, specialized sports applications, YouTube, Instagram, and TikTok. These platforms were used not only for content delivery but also for hosting interactive challenges, quests, and flash mobs (Table 1).

**Table 1:** Digital tools and their functional roles in the physical education curriculum.

Platform	Role in the educational process
Moodle/Google Forms	Assessment, reporting, and knowledge control
YouTube/Instagram	Video tutorials and exercise demonstrations.
TikTok	Interactive challenges, quests, and flash mobs
Specialized Sports Apps	Activity tracking and self-monitoring.

#### 2.2.4 Assessment and engagement

Students’ progress was monitored through digital quizzes, student-led exercise demonstrations, and self-control logs. To foster autonomy, participants were encouraged to perform daily morning routines and evening self-study sessions, earning additional points for theoretical homework and creative tasks.

### 2.3 Procedure

#### 2.3.1 Study design and timeline

The study was conducted from September to December 2025. The experimental design employed a two-stage testing protocol to assess participants’ physical activity, morpho-functional status, and psychological state. Baseline measurements were recorded on the first day of the experiment before the intervention. Post-test measurements were performed under identical conditions following the conclusion of the 15-week program. Consistent testing methodologies were applied across both stages to minimize systematic measurement error.

#### 2.3.2 Assessment of physical activity

Physical activity levels were evaluated using the International Physical Activity Questionnaire-Short Form (IPAQ-SF; Booth, 2000; Lee et al., 2011). This instrument assessed the intensity of physical activity and sedentary behavior (sitting time), enabling estimation of total physical activity in MET-min/week. 1 MET (metabolic equivalent of task) is equivalent to the consumption of 3.5 mL of oxygen per kilogram of body weight per minute, which approximates 1 kcal/kg/h at rest. Data collection was facilitated via an electronic survey created in Google Forms. Following the IPAQ-SF protocol, respondents were categorized into low, medium, or high physical activity levels.

#### 2.3.3 Morpho-functional status

The evaluation of morpho-functional status focused on the Body Mass Index (BMI). Participants received standardized instructions for self-reported anthropometric measurements at home, including requirements for scale placement on hard surfaces and height measurement against a vertical wall without footwear. BMI was calculated using the Quetelet formula via a specialized online tool (Online BMI, n.d.).

$$\mathrm{BMI}=m\;(\mathrm{kg})/h^{2}\;(m)$$

#### 2.3.4 Psychological state assessment

The “Well-being, Activity, and Mood” (WAM) questionnaire was used to assess psychological states (SAN [Well-being, Activity, and Mood], n.d.). The tool was digitized via Google Forms and administered during synchronous Zoom sessions to capture participants’ immediate states during the educational process. Following the WAM methodology, participants rated 30 pairs of bipolar adjectives (e.g., active–passive, cheerful–depressed) on a 7-point scale (3-2-1-0-1-2-3). These items correspond to three dimensions: Wellbeing (strength, health, fatigue); Activity (mobility, speed, and tempo); and Mood (emotional state and morale). For statistical processing, raw scores were converted to a 1–7 scale, with 1 representing the most negative state and 7 the most positive. Mean scores were calculated for each dimension and as a cumulative total. Data were automatically aggregated via Google Forms and integrated into the Moodle platform for comprehensive profile analysis. The internal consistency of the WAM questionnaire within the present sample was highly satisfactory, yielding Cronbach’s alpha (*α*) coefficients of 0.82 for Wellbeing, 0.79 for Activity, and 0.85 for Mood scales, indicating robust reliability. Previously, questionnaire validity was also verified using Cronbach’s alpha test (*α* = 0.81; Malinauskas and Vaicekauskas, 2013).

### 2.4 Statistical analysis

Statistical analysis of the research results was conducted using Microsoft Excel (version 2016; Microsoft, Redmond, WA, USA) and SPSS Statistics (Version 22.0; IBM Corp., Armonk, NY, USA). Descriptive statistics, such as the arithmetic mean *(X̄)* and standard deviation *(s)*, were calculated for all studied parameters. The data’s normality was checked with the *Kolmogorov–Smirnov test.* To assess the effectiveness of the 15-week intervention within a single experimental group (*n =* 735), a comparison between baseline (pre-test) and final (post-test) measures was performed. The total group indicators were calculated by aggregating the data from both female (*n =* 374) and male (*n =* 361) sub-samples to assess the overall impact of the program. The significance of any changes was tested using the *paired-samples t-test.* The percentage change (%) between pre- and post-intervention stages was also calculated. A *p*-value < 0.05 was regarded as statistically significant.

To evaluate the practical significance of the findings, effect sizes were calculated using Cohen’s *d* for paired samples, interpreted as small (0.20), medium (0.50), or large (0.80). Additionally, 95% confidence intervals (95% CI) were calculated for all mean differences.

## 3 Results

The effectiveness of a 15-week experimental training program was evaluated by comparing baseline and final metrics of one experimental group (*n =* 735; Table 2).

**Table 2:** Dynamics of physical and psychological indicators of students during the intervention (*n* = 735).

Parameters		Gender	*n*	Pre-test (*X̄* ± *s*)	Post-test (*X̄* ± *s*)	Δ%	*t*	*p*	95% CI	Cohen’s *d*
BMI (kg/м^2^)		Female	374	26.33 ± 3.28	26.29 ± 3.28	−0.15	2.21	0.058	[−0.08, 0.00]	0.01
		Male	361	24.56 ± 3.35	24.58 ± 3.35	+0.02	2.39	0.056	[0.00, 0.04]	0.01
		Total	735	25.46 ± 3.31	25.44 ± 3.31	−0.08	1.23	0.219	[−0.05, 0.01]	0.01
IPAQ-SF (МЕТ-min/week)		Female	374	3,617.03 ± 271.76	3,637.18 ± 215.77	+0.55	1.73	0.030	[1.82, 38.48]	0.08
		Male	361	3,557.03 ± 284.76	3,575.18 ± 212.77	+0.50	1.83	0.050	[−1.34, 37.64]	0.07
		Total	735	3,587.56 ± 278.13	3,606.74 ± 214.30	+0.53	2.52	0.012	[5.11, 33.25]	0.08
WAM (points)	Wellbeing	Female	374	30.14 ± 2.96	30.38 ± 2.91	+0.78	1.49	0.039	[0.01, 0.47]	0.08
		Male	361	32.87 ± 2.96	33.22 ± 2.91	+1.05	2.04	0.013	[0.01, 0.69]	0.12
		Total	735	31.48 ± 2.96	31.77 ± 2.91	+0.92	2.50	0.013	[0.06, 0.52]	0.10
	Activity	Female	374	46.09 ± 2.58	47.15 ± 2.14	+2.24	2.37	0.024	[0.18, 1.94]	0.45
		Male	361	46.92 ± 2.58	47.08 ± 2.14	+0.33	1.38	0.411	[−0.07, 0.39]	0.07
		Total	735	46.50 ± 2.58	47.12 ± 2.14	+1.33	2.66	0.008	[0.16, 1.08]	0.26
	Mood	Female	374	47.02 ± 2.99	48.22 ± 2.99	+2.40	4.23	0.001	[0.64, 1.76]	0.40
		Male	361	50.72 ± 2.99	51.08 ± 2.99	+0.70	1.55	0.050	[0.00, 0.72]	0.12
		Total	735	48.84 ± 2.99	49.60 ± 2.99	+1.56	4.11	0.001	[0.40, 1.12]	0.25

*X̄*, arithmetic mean; *s*, standard deviation; BMI, Body Mass Index; IPAQ-SF, International Physical Activity Questionnaire-Short Form; WAM, Wellbeing, Activity, Mood Questionnaire; *n*, sample; Δ%, percentage of change between pre- and post-intervention; *t*, Student’s *t*-test; *p*, significance level; 95% CI, 95% confidence interval for mean difference; Cohen’s *d*, standardized effect size.

The analysis of morphofunctional indicators showed that during the intervention period, participants’ BMI did not change significantly, which is typical of short-term programs. Female students showed a marginal trend toward a decrease in BMI by 0.15% (*p* = 0.058), while male students demonstrated a minimal increase of 0.02% (*p* = 0.056). Overall, the BMI index for the entire sample showed notable stability with a non-significant decrease of only 0.02 kg/m^2^ (0.08%).

At the same time, the level of physical activity, as measured by the IPAQ-SF questionnaire, showed positive dynamics across both gender subgroups. Female students increased their energy expenditure by 20.15 MET-min/week (*p* = 0.030), whereas male students demonstrated an increase of 18.15 MET-min/week (*p* = 0.050). Overall, the entire sample exhibited a statistically significant increase in energy expenditure of 19.18 MET-min/week (*p* = 0.012). This indicates that the proposed program demonstrated potential in counteracting hypodynamia, supporting students’ ability to maintain or incrementally increase their energy expenditure even under distance learning conditions.

The most notable trends during the program implementation were found in the psycho-emotional sphere (WAM test), with the positive changes being especially pronounced among female students. Specifically, the mood indicator among female participants demonstrated a statistically significant increase of 1.20 points (*p* = 0.001), suggesting a positive trend toward emotional stabilization during the intervention. In male students, the mood increase was 0.36 points (*p* = 0.050). Furthermore, female students showed a significant improvement in their activity scores by 1.06 points (*p* = 0.024), whereas the changes among their male peers did not reach statistical significance (*p* = 0.411). Conversely, male students demonstrated a slightly more pronounced improvement in wellbeing by 0.35 points (*p* = 0.013) compared to an increase of 0.24 points observed in female students (*p* = 0.039). Overall, positive dynamics were observed across the entire sample on all scales, with incremental improvements in the total scores for wellbeing (+0.92%, *p* = 0.013), activity (+1.33%, *p* = 0.008), and mood (+1.56%, *p* = 0.001).

## 4 Discussion

The World Health Organization emphasizes that regular and adequate physical activity is critical for promoting both physical and mental health in young people (WHO, 2022). However, current evidence indicates a global physical inactivity deficit among students (Aubert et al., 2022; Kolomiitseva et al., 2020; Li et al., 2023; López-Valenciano et al., 2021). In Ukraine, this problem has become particularly acute due to the combined impact of the COVID-19 pandemic and ongoing military conflict, which has led to a prolonged transition to online learning, especially at universities located close to the war zone (Griban et al., 2024; Muntian and Poproshaiev, 2024; Zakomorna et al., 2024). Research confirms that such isolation and physical inactivity are correlated with increased depression and worsening psycho-emotional wellbeing in students (Li et al., 2023; Morres et al., 2021; Rak et al., 2024).

The results of our study demonstrate that integrating traditional physical exercise, communication, and digital technologies into physical education effectively counteract these negative trends. We thus agree with the opinions of Bouchey et al. (2021), Cabral et al. (2022), Li and Lim (2025), Shepelenko et al. (2023), and the WHO report (WHO, 2025a) that integrated approaches and hybrid technologies positively impact learning outcomes.

Despite the challenges inherent in the remote-learning format, the study demonstrated positive trends across key indicators. The integration of synchronous Zoom sessions and flexible asynchronous tools (Moodle, YouTube, social media, health calculators, and smart applications) was associated with a statistically significant increase in students’ energy expenditure. This aligns with previous findings (Li et al., 2023; Shepelenko et al., 2023; Petrova and Latyshev, 2025) suggesting that digital platforms and innovative pedagogical approaches serve as viable tools for supporting physical activity within restricted environments. Specifically, female students demonstrated an increase of 20.15 MET-min/week (*p* = 0.030, Cohen’s *d* = 0.08), while male students showed an increase of 18.15 MET-min/week (*p* = 0.050, Cohen’s *d* = 0.07). Although a mean increase of 18–20 MET-min/week may appear modest under normal baseline conditions, within the context of active conflict, localized movement restrictions, and severe displacement, this stability and incremental growth represent a critical behavioral shift. Statistical analysis confirmed that even this marginal improvement significantly correlated with enhanced psycho-emotional resilience and mitigated the risks of acute hypokinesia among the participants.

Overweight and obesity remain pressing public health challenges globally (WHO, 2025b). A primary contributor to these issues is physical inactivity, which often stems from restricted mobility and reduced social engagement (Kolomiitseva et al., 2020; WHO, 2025b). In our sample, however, BMI values remained statistically stable during the intervention. Although existing literature frequently notes a trend toward increased BMI during periods of prolonged online learning (Xia et al., 2021; Hao et al., 2025), our data revealed a nominal, non-significant decrease of 0.08% (from 25.46 to 25.44 kg/m^2^, *p* = 0.219). This trend suggests that the flexible, digitally oriented approach may have supported students in mitigating the excess weight gain typically associated with a sedentary lifestyle. Interestingly, the baseline BMI profiles of these Ukrainian undergraduate students, the majority of whom fell within the normal range, align with earlier comparative findings (Bergier et al., 2018).

We acknowledge that while changes in physical activity levels reached statistical significance due to the substantial sample size (*n* = 735), the practical and clinical magnitude of both BMI and MET-min/week shifts remains modest, as indicated by the negligible to small effect sizes. Nevertheless, within the specific context of wartime displacement, localized movement restrictions, and acute stress, maintaining baseline BMI stability and preventing a downward trend in physical activity can be interpreted as a meaningful and practical outcome for health promotion in crisis settings.

The program’s most significant effect was observed in psychological wellbeing (WAM test). Improvements were observed across all scales: wellbeing (+0.92%), activity (+1.33%), and mood (+1.56%). The increase in mood scores was particularly pronounced among female students (*p* = 0.001). This is consistent with data showing that organized physical activity promotes self-regulation and psychological resilience, which is critical for students living in frontline regions under constant stress (Joseph, 2025; Mahindru et al., 2023; Petrova and Latyshev, 2025; Rak et al., 2024).

Regarding gender differences, female students showed more pronounced improvements in mood (+1.2 points, *p* = 0.001) and activity than male peers. This pattern may be explained by differences in coping mechanisms; the literature suggests that female individuals often use structured group fitness activities and interactive digital socialization (such as the implemented social media challenges and quests) as effective tools for emotional expression and anxiety mitigation (Sheng et al., 2025). Conversely, male students may be more oriented toward competitive or high-intensity resistance training, which is harder to fully experience or replicate in a confined remote learning environment (Espada et al., 2023).

Testing an experimental physical education teaching method among first-year students demonstrated short-term (15 weeks) positive dynamics in their physical activity levels, functional fitness, and psycho-emotional state. The findings are consistent with the conclusions of several recent studies (Li et al., 2023; Shepelenko et al., 2023; Tao et al., 2024). However, the sustainability of these effects over the long term remains unclear, necessitating further study and, potentially, modification of the intervention strategy to prolong the achieved results (Cabral et al., 2022; Olson and McAuley, 2015).

It is important to note that our study has certain limitations, particularly the absence of a control group due to organizational challenges and students’ unequal access to safe physical activity amid martial law. Nevertheless, the large sample size (*n =* 735) and statistically significant changes observed in both gender subgroups and the overall sample suggest that our proposed method holds potential for supporting students’ physical activity and psycho-emotional wellbeing under crisis conditions. Our findings complement existing research (Da-Wei et al., 2018; Petrova and Latyshev, 2025; Shepelenko et al., 2023; Valencia Pilamunga et al., 2026; Yefremenko et al., 2025) regarding the utility of hybrid, integrated approaches in pedagogy, and underscore the value of further development of adaptive digital strategies in physical education.

A primary methodological limitation is the reliance on self-reported data, including anthropometric metrics, the IPAQ-SF questionnaire, and the WAM inventory, which may introduce inherent measurement or recall biases. We acknowledge that laboratory monitoring of objective physiological biomarkers was unfeasible due to the inherent constraints of the distance-learning format. However, standardized self-assessment tools like the WAM questionnaire are well-established, validated indicators of psycho-emotional dynamics. Furthermore, digital quizzes were not employed to measure physical fitness directly, but rather to evaluate the cognitive component of physical literacy, which underpins long-term health behavior changes.

Additionally, due to the extreme conditions of the wartime period, it was impossible to fully control for critical confounding variables, such as fluctuating daily stress levels, alterations in dietary habits, and diverse socioeconomic or living conditions resulting from displacement. Future studies conducted under more stable conditions should aim to integrate objective monitoring tools, such as wearable fitness trackers, alongside comprehensive lifestyle logs to mitigate these confounding factors and further validate the utility of adaptive digital strategies in physical education.

## 5 Conclusion

The introduction of a digitally oriented approach to basic physical education, combining synchronous (Zoom) and asynchronous (Moodle, YouTube, social media) digital teaching methods with traditional physical exercises (gymnastics, aerobics, Pilates, Tabata, etc.), represents a viable and promising framework in the context of extended distance learning. Our findings indicate that this approach is associated with the mitigation of physical inactivity, supporting students’ ability to maintain or incrementally increase their physical activity levels amid social isolation and the severe challenges associated with martial law. The integration of diverse digital tools and structured physical exercises demonstrates the potential to facilitate the development of regular exercise habits and compensate for acute hypokinesia, regardless of students’ geographic location. Structured physical activity, delivered in interactive digital formats, may serve as an important supportive instrument for enhancing psycho-emotional resilience, potentially contributing to the reduction of stress and anxiety during prolonged crises.

These observed trends suggest that this multimodal program, which complements basic physical training with digital integration, could be considered for wider piloting and implementation in higher education institutions forced to operate remotely or in blended formats.

## Data Availability

The raw data supporting the conclusions of this article will be made available by the authors, without undue reservation.
